# An ovine knee simulator: description and proof of concept

**DOI:** 10.3389/fbioe.2024.1410053

**Published:** 2024-06-27

**Authors:** Maria Kristina Bartolo, Simon Newman, Oliver Dandridge, Camilla Halewood, Mario Alberto Accardi, Daniele Dini, Andrew A. Amis

**Affiliations:** ^1^ Biomechanics Group, Mechanical Engineering Department, Imperial College London, London, United Kingdom; ^2^ Orthonika Ltd, London, United Kingdom; ^3^ Department of Surgery and Cancer, Imperial College London School of Medicine, London, United Kingdom

**Keywords:** knee, meniscus replacement, ovine gait simulator, kinematics, contact pressures

## Abstract

**Aims:**

The ovine stifle is an established model for evaluation of knee treatments, such as meniscus replacement. This study introduces a novel ovine gait simulator for pre-testing of surgical treatments prior to *in vivo* animal trials. Furthermore, we describe a pilot study that assessed gait kinematics and contact pressures of native ovine stifle joints and those implanted with a novel fiber-matrix reinforced polyvinyl alcohol-polyethylene glycol (PVA-PEG) hydrogel meniscus to illustrate the efficacy of the simulator.

**Methods:**

The gait simulator controlled femoral flexion-extension and applied a 980N axial contact force to the distal tibia, whose movement was guided by the natural ligaments. Five right ovine stifle joints were implanted with a PVA-PEG total medial meniscus replacement, fixed to the tibia via transosseous tunnels and interference screws. Six intact and five implanted right ovine stifle joints were tested for 500 k gait cycles at 1.55 Hz. Implanted stifle joint contact pressures and kinematics in the simulator were compared to the intact group. Contact pressures were measured at 55° flexion using pressure sensitive film inserted sub-meniscally. 3D kinematics were measured optically across two 30-s captures.

**Results:**

Peak contact pressures in intact stifles were 3.6 ± 1.0 MPa and 6.0 ± 2.1 MPa in the medial and lateral condyles (*p* < 0.05) and did not differ significantly from previous studies (*p* > 0.4). Medial peak implanted pressures were 4.3 ± 2.2 MPa (*p* > 0.4 versus intact), while lateral peak pressures (9.4 ± 0.8 MPa) were raised post medial compartment implantation (*p* < 0.01). The range of motion for intact joints was flexion/extension 37° ± 1°, varus/valgus 1° ± 1°, external/internal rotation 5° ± 3°, lateral/medial translation 2 ± 1 mm, anterior/posterior translation 3 ± 1 mm and distraction/compression 1 ± 1 mm. Ovine joint kinematics in the simulator did not differ significantly from published *in vivo* data for the intact group, and the intact and implanted groups were comparable (*p* > 0.01), except for in distraction-compression (*p* < 0.01).

**Conclusion:**

These findings show correspondence of the ovine simulator kinematics with *in vivo* gait parameters. The efficacy of the simulator to evaluate novel treatments was demonstrated by implanting a PVA-PEG hydrogel medial meniscal replacement, which restored the medial peak contact pressures but not lateral. This novel simulator may enable future work on the development of surgical procedures, derisking subsequent work in live animals.

## 1 Introduction

Pre-clinical assessment of novel orthopaedic joint procedures generally includes material characterization and biomechanical testing in the lab setting, biocompatibility evaluation and *in vivo* studies in large animal models prior to the commencement of clinical trials. Biomaterial characterization provides insight into the properties and performance of single or combined materials using physiologically relevant parameters but is limited by sample geometry and controlled environments. *In vivo* implantation in large animal models such as goats or sheep (Zur et al., 2011; Patel et al., 2015; Vrancken et al., 2017) provides a thorough evaluation of the design, safety, performance and biocompatibility of the novel procedure *in vivo*, thus derisking the procedure before clinical trials. However, such studies are costly and time-consuming, and researchers are ethically bound to reduce such studies and limit sample sizes as far as possible while satisfying regulatory requirements.

The ovine stifle joint is a well-established knee joint model for pre-clinical research in reconstruction or replacement of the cruciate and collateral ligaments, partial and total meniscus replacements, cartilage lesion repair and osteoarthritis treatment (Radford et al., 1996; Tapper et al., 2006; Taylor et al., 2006; Zur et al., 2011; Gregory et al., 2012; Patel et al., 2015)

A clear gap exists in the pre-clinical assessment of novel orthopaedic joint procedures, for dynamic cadaveric testing of the ovine knee joint under simulated physiological gait, as a screening method and at a reduced cost (Maher et al., 2011), before advancing to *in vivo* animal studies. Research studies have developed techniques for dynamic testing of meniscus replacements in cadaveric ovine stifles but were limited to low axial loads and were only run for 10 cycles (Cottrell et al., 2008; Brophy et al., 2010; Holloway, 2012). This paper describes such a simulator, its verification in terms of loading and kinematics, then a proof of concept study in which a meniscus replacement is tested in a cadaveric ovine knee joint under simulated gait.

Meniscal surgery is one of the most common orthopaedic surgical interventions (Englund and Lohmander, 2006; McDermott et al., 2008; Abram et al., 2018). Although meniscectomy is the current standard of care after irreparable meniscal injury, and may alleviate symptoms in the short-term, it increases the risk of the onset of osteoarthritis (Englund and Lohmander, 2006; McDermott et al., 2008). In such cases, high tibial osteotomy is often performed to shift joint contact loads to the unaffected compartment in order to reduce pain and help slow OA progression in the meniscectomized compartment (Lee and Byun, 2012). Meniscal allograft transplants (Novaretti et al., 2019; Verdonk et al., 2013) and partial replacement scaffolds (Stone et al., 1997; Verdonk et al., 2011) have been used to replace the injured meniscus, with limited success in long-term function and survivorship (Hutchinson et al., 2013; Winkler et al., 2020). Novel total meniscus replacement devices aim to fill this treatment gap (Zur et al., 2011; Patel et al., 2015; Vrancken et al., 2017). No proven long-term joint-preserving treatment options exist for patients with irreparable meniscal damage.

The aim of this study was to develop an ovine knee joint simulator and to assess the kinematics and contact pressures of the ovine stifle joint under simulated gait. The use of the simulator will be demonstrated in a pilot study when the knee has been implanted with a novel fiber-matrix reinforced polyvinyl alcohol-polyethylene glycol (PVA-PEG) hydrogel meniscus and to compare it against intact stifles. It was hypothesized that the gait kinematics and contact pressures post meniscal replacement would not differ significantly from those of intact stifles.

## 2 Materials and methods

**FIGURE 1:**
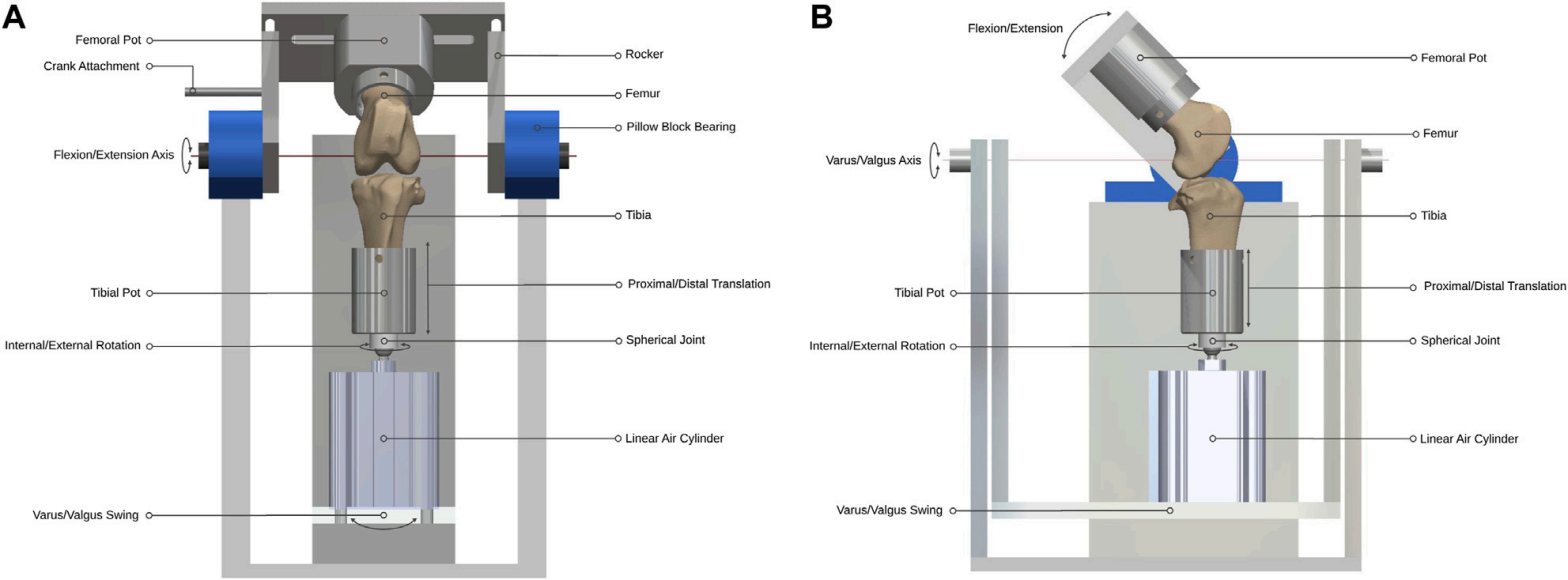
Ovine gait simulator joint assembly. **(A)** A front and **(B)** side view schematic of the joint assembly.

Two single-station ovine gait simulators were used (Figure 1). The joint simulator controlled femoral flexion-extension and applied a cyclic axial contact force to the distal end of the tibia, while allowing articular movement in five degrees of freedom (DOF). During knee flexion-extension, tibial movement was guided by the natural ligaments of the stifle joint, namely, the anterior (cranial) cruciate ligament, the posterior (caudal) cruciate ligament and the medial and lateral collateral ligaments, which were left intact together with surrounding soft tissue structures. This method was expected to result in stifle kinematics that were not significantly different from *in vivo* conditions because motion of the natural knee is restricted and stabilized by passive soft tissues (Houtem et al., 2006; Taylor et al., 2006). The simulator design was based on a force-controlled joint simulator that has been shown to allow physiological movement of cadaveric knee joints under gait loading conditions (Houtem et al., 2006).

The gait simulator ran at a frequency of 1.55 Hz for 500,000 gait cycles (3.73 days), equivalent to approximately a year of normal use by adult sheep *in vivo* (Ruckstuhl, 1998). The simulator was mounted inside a refrigerator to maintain the stifle at 4°C–8°C to delay tissue necrosis for the duration of the test. An in-line pressure syringe pump (Graseby 3200; Smiths Medical International, MN) was used to deliver diluted sterile-filtered Bovine Calf Serum (BCS) (product 12133C; Merck Group, Germany) from a mounted 50 mL Luer Lock syringe at a rate of 2 mL per hour. The lubricant was delivered directly into the joint space throughout the test by placing an 18G × 38 mm cannula in the femoral intercondylar notch. The syringe pump was replenished with fresh lubricant every 24 h.

### 2.1 Ovine gait simulator design

**FIGURE 2:**
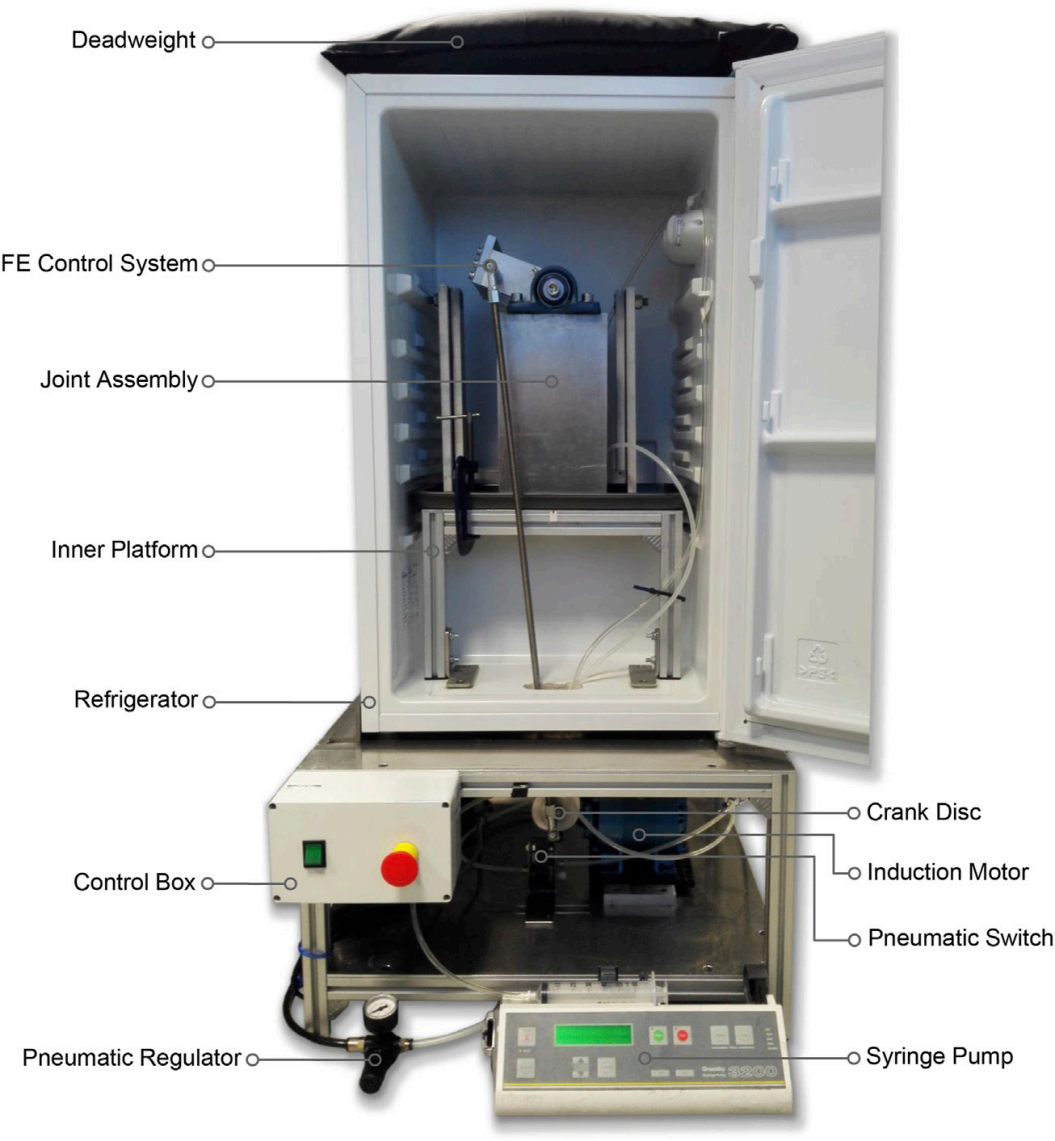
Ovine gait simulator assembly.

Design parameters for the joint simulator were determined from *in vivo* ovine biomechanics literature (Table 1). The overall configuration of the simulator had the femoral mounting driven in flexion-extension in a vertical plane while the tibial mounting was quasi-static below it. A crank-rocker mechanism based on Grashof’s theorem imposed the flexion-extension of the femoral mounting within the physiological walking range of 45–80 degrees flexion (Tapper et al., 2006). All other femoral DOF were constrained. Flexion-extension of the femoral mounting about the transepicondylar axis was allowed through self-aligning bearings (Pillow Block Bearing; RS Components, England) and controlled by a connecting rod driven by a rotating crank disc (Figure 2). The crank disc was mounted onto a single-speed induction geared motor (model SD18-0083/CONT; Parvalux Electric Motors, England) with an output speed of 93 rpm, equivalent to 1.55 Hz. This provided a simplified approximation of the gait cycle, but with a shorter stance phase (34% of the gait cycle) than that observed *in vivo*.

**TABLE 1:** Design parameters of the ovine gait simulator.

Design Parameter	Design Specification	Reference Values	Source
Flexion-Extension (°)	Minimum: 45° Maximum: 80° Range: 35°	Average Min: 42°–49° Average Max: 70°–77° Range: 34° ± 5.2°	Tapper et al. (2004), Tapper et al. (2006), Taylor et al. (2006)
Peak Joint Axial Load in body weight (BW)	2BW ≡ 1 kN^a^	2.27 ± 0.44 BW	Taylor et al. (2011), Taylor et al. (2006)
Stance (° Flexion)	45°–55° Flexion	45°–55° Flexion	Tapper et al. (2004), Tapper et al. (2006), Taylor et al. (2006)
Stance (% gait)	34%	62.9% ± 4.08%	Tapper et al. (2004), Tapper et al. (2006), Taylor et al. (2006), Agostinho et al. (2012)
Gait Frequency (Hz)	1.55 Hz	0.85–2.27 Hz	Tapper et al. (2004), Tapper et al. (2006), Agostinho et al. (2012)

^a^ Based on a live animal weight of 55 kg.

The distal end of the tibia was mounted on a spherical joint, in line with the tibial long mechanical axis by a snug-fitting intramedullary rod to which the sphere was attached, that allowed unconstrained internal-external rotation with minimal resistance. Proximal and distal translation of the tibia were possible through the active and passive movement of the one-way linear air cylinder (model CDQ2B63TF-50DZ; SMC, England) piston rod during the stance and swing phases respectively (Figure 1). The linear air cylinder was fixed to a slotted swing allowing varus-valgus rotation of the tibia about an axis passing through the center of the tibiofemoral joint and perpendicular to the flexion-extension axis. The medial-lateral and anterior-posterior translations in the stifle joint are small (Tapper et al., 2006) and were accounted-for by small rotations of the spherical joint.

**FIGURE 3:**
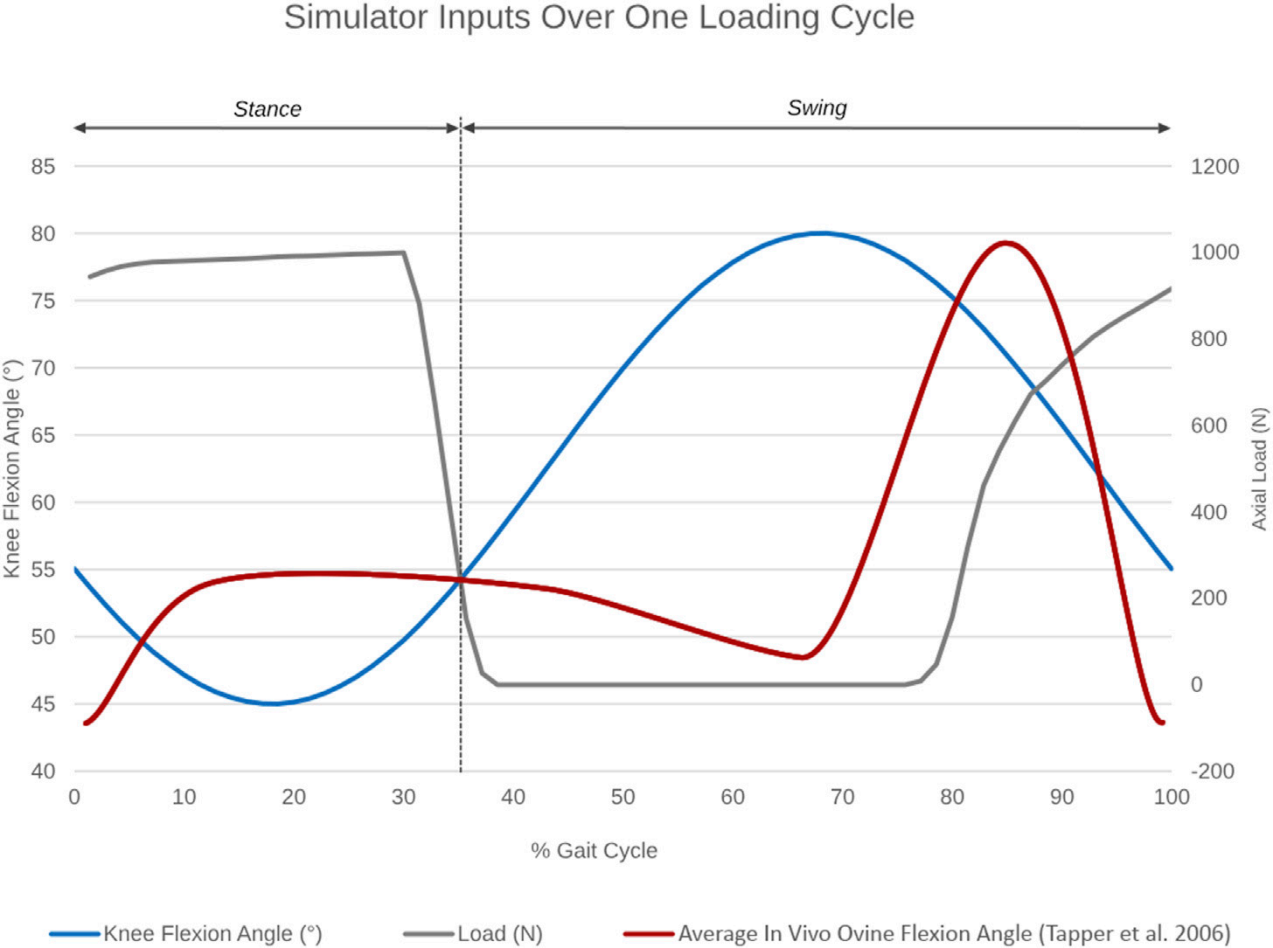
Simulator inputs as a function of percentage gait cycle over one loading cycle. Knee flexion angle ranges between 45°–55° during the stance and between 55°–80° during the swing phase. Axial load actuation starts at around 80% of the simulated gait cycle to reach a peak axial load of 980N in the subsequent stance phase. The average *in vivo* ovine flexion-extension angle over one gait cycle, as reported by Tapper et al. (2006), is superimposed in red for reference.

A cam profile, incorporated onto the crank disc, mechanically activated a roller lever pneumatic switch (model VM430-01-01, SMC, England) and controlled the gait loading profile synchronous with the flexion-extension motion of the simulator (Figure 3). The pneumatic switch actuated the linear air cylinder, which applied the axial contact force through the tibial shaft. Axial load was actuated at around 80% of the simulated gait cycle to reach a peak load of 980N in the subsequent stance phase. The peak axial load was held throughout the stance phase and then ramped down for the swing phase.

### 2.2 Specimen preparation

Six paired and five right fresh-frozen North of England Mule stifle joints (*n* = 17) of ewes aged >2 years and weighing 56–68 kg were used. Of the six paired stifles, the right stifles were tested as intact joints in the simulator and the left contralateral stifles remained untested as controls. Each right stifle was carefully dissected to preserve the surrounding muscle tissues, capsule, collateral and cruciate ligaments and patella. The femoral and tibial shafts were transected approximately 60 mm from the proximal end of the trochlear groove and 65 mm distally to the tibial tubercle, respectively. The transepicondylar axis of the femur was aligned with the flexion-extension axis of the simulator using a static alignment jig with 1.6 mm Kirschner wires that passed through the fixture along the flexion-extension axis so that their points engaged the epicondyles. The femur was then secured by three screws and potted in polymethyl methacrylate (PMMA) bone cement (Simplex Rapid, England). Correct alignment of the epicondylar axis was imperative for ensuring physiological kinematics of the stifle in the simulator (Bedi et al., 2010). The tibia was then centered in the base of the simulator and aligned such that the plateau was parallel to the ground at 55° flexion, the angle at peak load in mid-stance. The tibia was then secured using screws and bone cement. This protocol is analogous to knee joint alignment protocols adopted by similar studies (Cottrell et al., 2008; Bedi et al., 2010). Throughout this process, the stifle joint was kept moist with occasional water spray.

The five unpaired right stifle joints were prepared similarly and then implanted with the TMR device. Fiber-matrix reinforced PVA-PEG hydrogel meniscal replacement implants (*n* = 5) were designed and manufactured for the right medial stifle of the chosen breed and weight of sheep. A modified medial parapatellar arthrotomy was performed and then the medial collateral ligament (MCL) was released and reflected distally via an epicondylar osteotomy for adequate visualization of the medial compartment while preserving the natural ligament function and joint biomechanics (Kelly et al., 2007; Patel et al., 2015; Brzezinski et al., 2017). Following a total medial meniscectomy, the implant was inserted into the joint space and fixed to the tibia via transosseous tunnels and interference screws (Bartolo et al., 2022). The MCL was reattached securely by reducing and fixing the osteotomy with bicortical bone screws, then the joint was sutured closed.

### 2.3 Testing lubricant

As recommended by international joint replacement wear testing standards ISO 14243-3:2014 and ASTM F732-17 (BSI Standards Limited, 2014; ASTM International, 2017), stifle joints were lubricated with sterile-filtered BCS diluted with sterile deionized water to have a protein mass concentration of 20 g/L. A broad-spectrum biocide (ProClin 300; Merck Group, Germany) was added at a recommended concentration of 15 ppm to inhibit bacterial, fungal and yeast growth (SAFC Supply Solutions, 2008).

### 2.4 Dynamic cadaveric testing

Six intact and five implanted right stifle joints were tested in the simulator at a peak axial contact force of 980N for 500,000 cycles at a frequency of 1.55 Hz. The six contralateral stifle joints were left untested as controls for the survivability of the menisci and any signs of anomalous cartilage damage in the intact right stifle joints post-test. Each ovine gait simulator fatigue test was started immediately after the implantation of the TMR device in each cadaveric joint, to avoid the need for an additional freeze-thaw cycle of both the cadaveric joint and the device.

At the end of the test, stifle joint kinematics were recorded in the simulator using a motion-tracking system (Optotrak Certus; Northern Digital Inc., Canada). A three-marker rigid body was rigidly attached to each of the long bones using a bone pin. The markers were attached proximal to the femoral trochlear groove and on the proximal posteromedial aspect of the tibial shaft. A probe was used to digitize anatomic landmarks marked by small bone screws to define the joint coordinate system used clinically (Grood and Suntay, 1983). For the femur, the medial and lateral epicondyles and a central point at the proximal end of the bone shaft were digitized. For the tibia, the attachments of the medial and lateral collateral ligaments on the tibia and fibular head respectively, plus the center of the distal end of the bone shaft were digitized. Joint kinematics were captured on the simulator across two 30 s captures (93 strides) with a sampling frequency of 100 Hz.

Contact pressures on the medial and lateral condyles were measured statically at 55° flexion using pressure sensitive film (Prescale Low Pressure; Fujifilm, Japan) inserted under the native menisci or medial meniscus replacement implant. The surrounding soft tissues were removed, and a transverse capsulotomy was performed at the level of the tibial plateau, while preserving the cruciate and collateral ligaments, to allow the insertion of the film strip. Strips, approximately 25 mm wide and 120 mm long, were prepared as per the manufacturer’s instructions and then encased in plastic wrap to avoid fluid seepage (Bedi et al., 2010). Following strip insertion, an axial load of 980N was applied in the simulator, maintained for 5 s and then released. Three repeated measures were taken for each joint condyle. The strips with imprinted colour maps were scanned and converted to pressure maps using a specifically-developed machine learning model. The peak contact pressure, defined as the highest average pressure recorded per square 2 × 2 pixel area (equivalent to 0.28 mm^2^), was averaged across the three repeated measures for each joint. The position of the sensor was defined from the edge of the plateau.

Both untested left and tested right intact stifle joints were dissected to expose the menisci and the articulating cartilage surfaces by releasing the stabilizing ligaments and disarticulating the stifle. The meniscal and cartilage condition in untested joints and immediately post-test in tested intact joints was recorded photographically. In order to demonstrate cartilage surface damage such as fibrillation or erosion, the articular surfaces were then stained using black India ink (Meachim, 1972; Schmitz et al., 2010) and photographed.

### 2.5 Statistical analysis

Multiple *t*-tests were performed to determine statistical differences in the overall mean range of motion of the implanted group in each DOF when compared to the intact group and the *in vivo* kinematic data reported by Tapper et al. (2006) (*p* < 0.05). Statistical significance was determined and corrected for multiple comparisons using the Holm-Sidak method, with alpha = 0.01. Unpaired t-tests were performed to detect any statistical differences between the peak contact pressures recorded in each compartment of the implanted and intact groups (*p* < 0.05). Additionally, a paired *t*-test was performed to detect any significant differences between the peak medial and lateral contact pressures for the intact and implanted group separately (*p* < 0.05). Multiple t-tests were done to determine statistical differences between the measured peak medial and lateral contact pressures of the intact group to those recorded in literature (Lee-Shee et al., 2007; Heckelsmiller et al., 2017; Fischenich et al., 2018), and corrected for multiple comparisons using the Holm-Sidak method with an alpha = 0.05. Multiple t-tests were performed to determine any statistical differences in the measured peak medial and lateral contact pressures of the implanted group to the intact, meniscectomised, allograft and thermoplastic elastomer (TPE) hydrogel implanted groups reported by Fischenich et al. (2018), and corrected for multiple comparisons using the Holm-Sidak method with an alpha = 0.05. All analyses were conducted using GraphPad Prism (Version 8.4.3 for Windows, GraphPad Software, La Jolla, CA, United States).

## 3 Results

### 3.1 Joint kinematics

**FIGURE 4:**
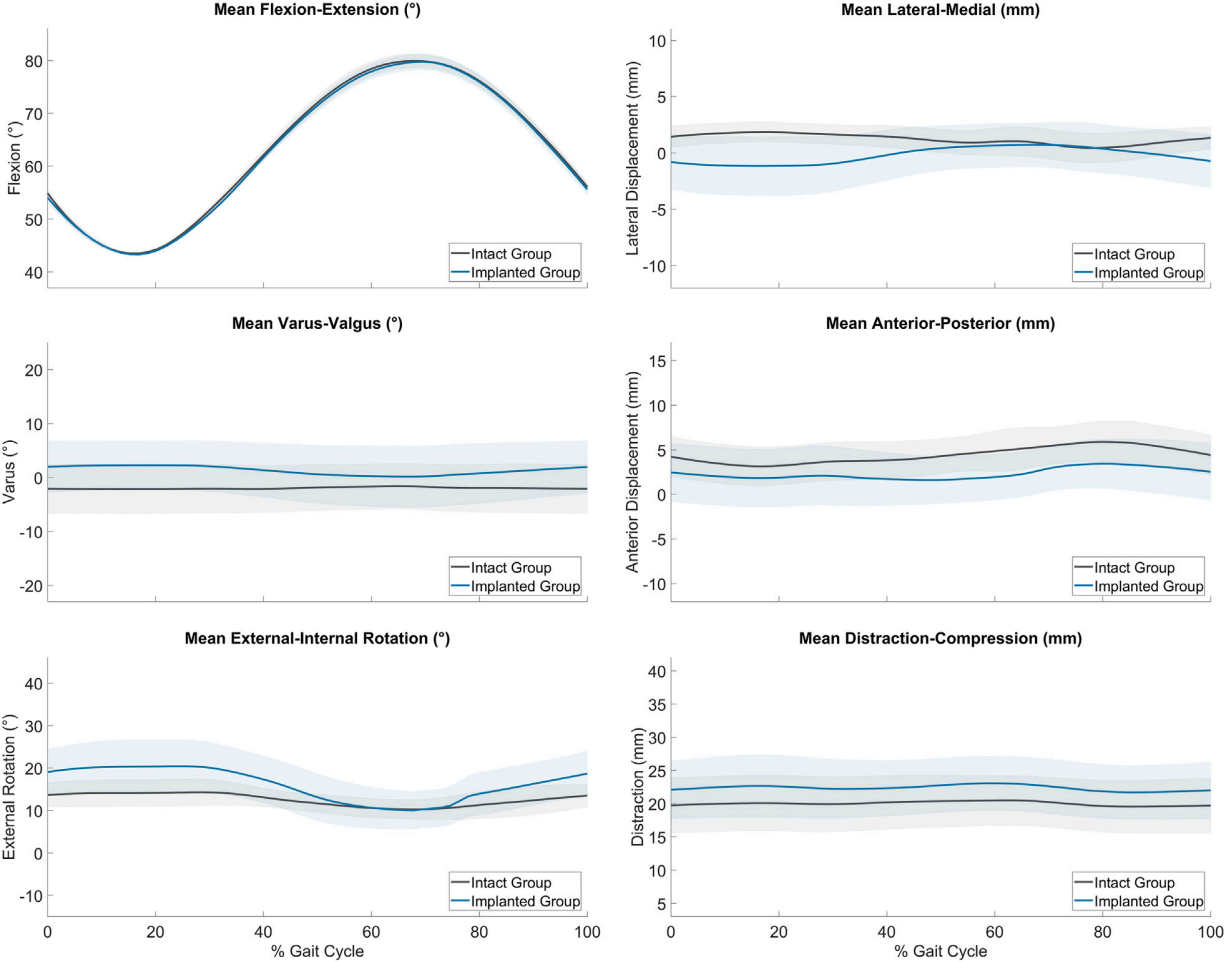
Average range of motion (± standard deviation) of the intact (grey, *n* = 6) and implanted (blue, *n* = 5) groups as measured on the ovine gait simulator.

The average joint kinematics versus percentage gait cycle in each of the six DOF were calculated for implanted joints and compared against the intact group as shown in Table 2 and Figure 4. The average range of motion of intact joints did not differ significantly from the kinematics recorded *in vivo* by Tapper et al. (*p* > 0.01 for distraction/compression, *p* > 0.1 for other ranges of motion) (Tapper et al., 2006). With increasing flexion, there was coupled tibial internal rotation and anterior and medial tibial translations in relation to the femur, with minimal translation and rotation in the remaining degrees of freedom. Inter-subject variability was lower than that recorded *in vivo*. The average range of motion of the implanted group did not differ significantly from the intact group for all degrees of freedom except for in distraction/compression (*p* < 0.01).

**TABLE 2:** The average range of motion (±standard deviation) of the intact and implanted groups in the gait simulator for the 6 DOF of the ovine stifle joint.

Group	Rotations (°)			Translations (MM)		
	Flexion/Extension	Varus/Valgus	External/Internal	Lateral/Medial	Anterior/Posterior	Distraction/Compression
INTACT (*n* = 6)	37 ± 1	1 ± 1	5 ± 3	2 ± 1	3 ± 1	1 ± 1
IMPLANTED (*n* = 5)	37 ± 1	3 ± 2	11 ± 4	2 ± 1	2 ± 1	3 ± 1
*p*-value	0.91	0.28	0.07	0.68	0.68	0.008^a^

^a^ Shows statistical significance between groups.

### 3.2 Survivability of native menisci and cartilage

No tissue damage or degeneration was observed on the medial and lateral menisci of tested joints with one exception. Slight degeneration was observed on the central inferior part of the medial meniscus of one right stifle. Comparable tissue damage was observed in the same location on the medial meniscus of the contralateral untested joint. No anomalous signs of cartilage damage were recorded on the tested joints in comparison to the untested contralateral joints following ink-staining.

### 3.3 Contact pressures

**FIGURE 5:**
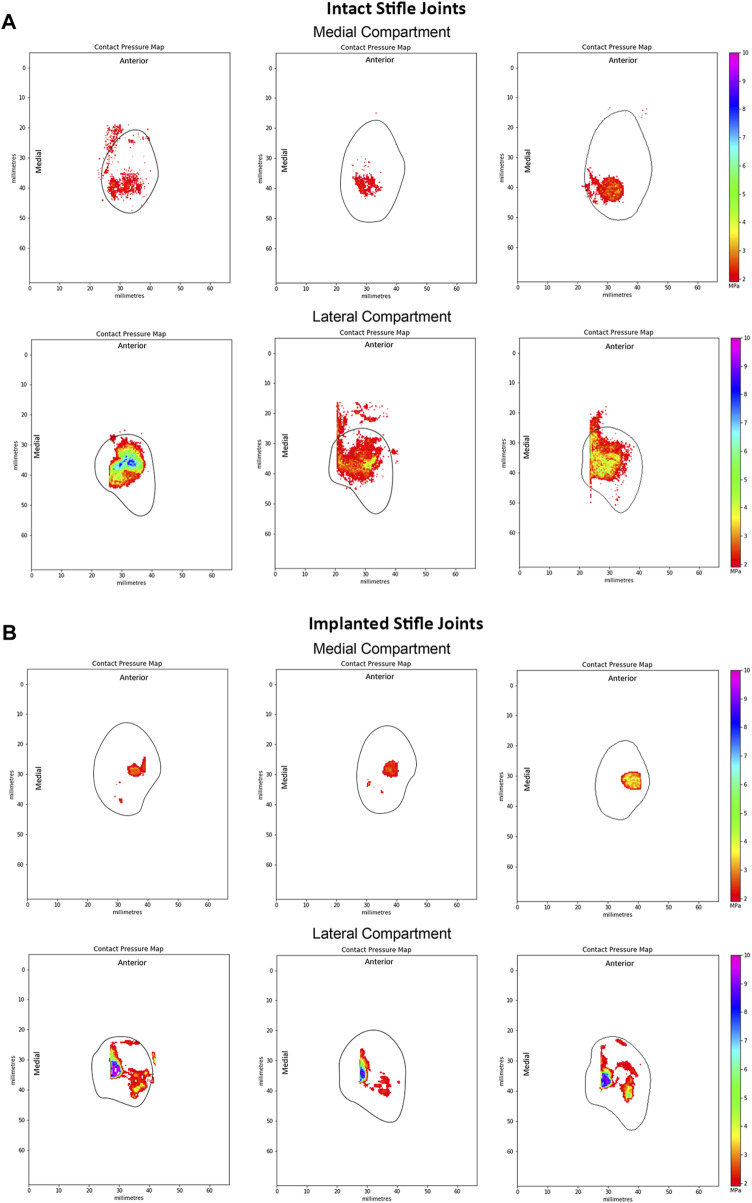
Representative peak contact pressure maps of the medial and lateral condyles for **(A)** intact joints and **(B)** implanted joints. Measured at 55° flexion under a 980N load in intact ovine stifle joints. The solid black line shows the approximate edge of the tibial plateau in each compartment of the knee.

Peak contact pressures recorded in intact stifles were 3.6 ± 1.0 MPa and 6.0 ± 2.1 MPa in the medial and lateral condyles respectively (*p* < 0.05). Peak pressures were generally located on the posterior aspect of the medial condyle covered by the meniscus and distributed across the central aspect of the lateral condyle (Figure 5A).

The measured static medial and lateral peak pressures of the intact group did not differ significantly from Fischenich et al. (2018) at 113 kg joint load at both 45° and 60° flexion and Heckelsmiller et al. (2017) (*p* > 0.4). Recorded medial peak pressures were significantly lower than those reported by Lee-Shee et al. (2007) (*p* < 0.05).

Following implantation in the medial compartment, the peak contact pressures were 4.3 ± 2.2 MPa and 9.4 ± 0.8 MPa in the medial and lateral condyles respectively, as shown in Figure 5B. Peak pressures were higher on the lateral condyle (*p* < 0.01). In the implanted ovine stifles, contact pressures were typically located on the central aspect of the medial condyle at the cartilage-to-cartilage contact area and distributed across the central aspect of the lateral condyle. Medial peak pressures were not significantly different between the implanted and intact groups (*p* > 0.4), while lateral peak pressures were significantly higher in the implanted group (*p* < 0.01).

## 4 Discussion

The most important findings of this study were that the novel ovine gait simulator led to peak contact pressures and kinematics of intact stifle joints that did not differ significantly from those reported in literature. The fiber-matrix reinforced PVA-PEG hydrogel medial meniscal replacement restored the medial peak contact pressures, but not the lateral contact pressures, thus demonstrating an example of the potential use of the simulator.

Previous research studies that performed fatigue testing of cadaveric ovine stifles for meniscus evaluation were limited to low axial contact loads, thus their results could not be compared to this study (Cottrell et al., 2008; Brophy et al., 2010; Maher et al., 2011; Holloway, 2012). To the authors’ knowledge, this study is the first to perform fatigue testing of ovine cadaveric joints under simulated physiologic gait conditions. The absence of macroscopic damage or degeneration on native menisci and their attachments, and comparable cartilage condition following 500,000 gait cycles, confirmed native tissue survivability during a test that was designed to replicate normal joint function. Future studies should assess the articular surfaces for damage after novel surgical procedures.

The peak contact pressures were of primary investigative interest for this study to confirm physiologic cartilage loading on each of the condyles. The static medial and lateral peak pressures in the intact group did not differ significantly from published literature (Heckelsmiller et al., 2017; Fischenich et al., 2018), which also reported higher lateral peak pressures. Contradictory results from Lee-Shee et al. (2007) show higher peak contact pressures on the medial condyle with a magnitude of 8 ± 1 MPa. The large variability in peak contact pressures recorded in literature can most likely be attributed to the varying contours of the stifle joint plateaux (Lee-Shee et al., 2007). However, the contrasting results from Lee-Shee et al. could be a result of the constraint of the varus-valgus and internal-external rotations of the joints during their testing. The results of this study may also provide a closer representation of the peak contact pressures in the joint given that Tekscan pressure mats, such as those used by Fischenich et al. and Heckelsmiller et al., are relatively stiffer making them less suitable and less repeatable in small and substantially curved surfaces such as those of the ovine stifle joint (Brand, 2005; Herregodts et al., 2015). Furthermore, Tekscan pressure mats have a lower spatial resolution than pressure sensitive films and require averaging across sensor nodes due to artefactual recordings (Brand, 2005). It remains possible that the peak pressures measured were inaccurate due to a combination of the contact area not being measured around the cruciate ligaments, and saturation of the pressure film in part due to shear loading effects from the sloping articular surface in that area.

Research studies have evaluated the peak contact pressures of meniscus replacement or scaffold implanted ovine stifle joints in comparison to the intact, partially or fully meniscectomized conditions (Brophy et al., 2010; Maher et al., 2011; Holloway, 2012; Fischenich et al., 2018). The implanted results from the present study were directly compared to those reported by Fischenich et al. for the intact, meniscectomized, allograft and TPE hydrogel implanted conditions performed in the medial compartment at 60° flexion with an axial load of 893N (91 kg) (Fischenich et al., 2018). All other studies were limited to the lateral compartment and low axial loads. The medial and lateral peak pressures were the primary interest for this study, to provide insight on the load bearing and load transmission capabilities of the implant in comparison to the intact condition during loading that simulated normal ovine gait and, thus, showed the usefulness of the simulator as a screening tool for total meniscus replacements.

**FIGURE 6:**
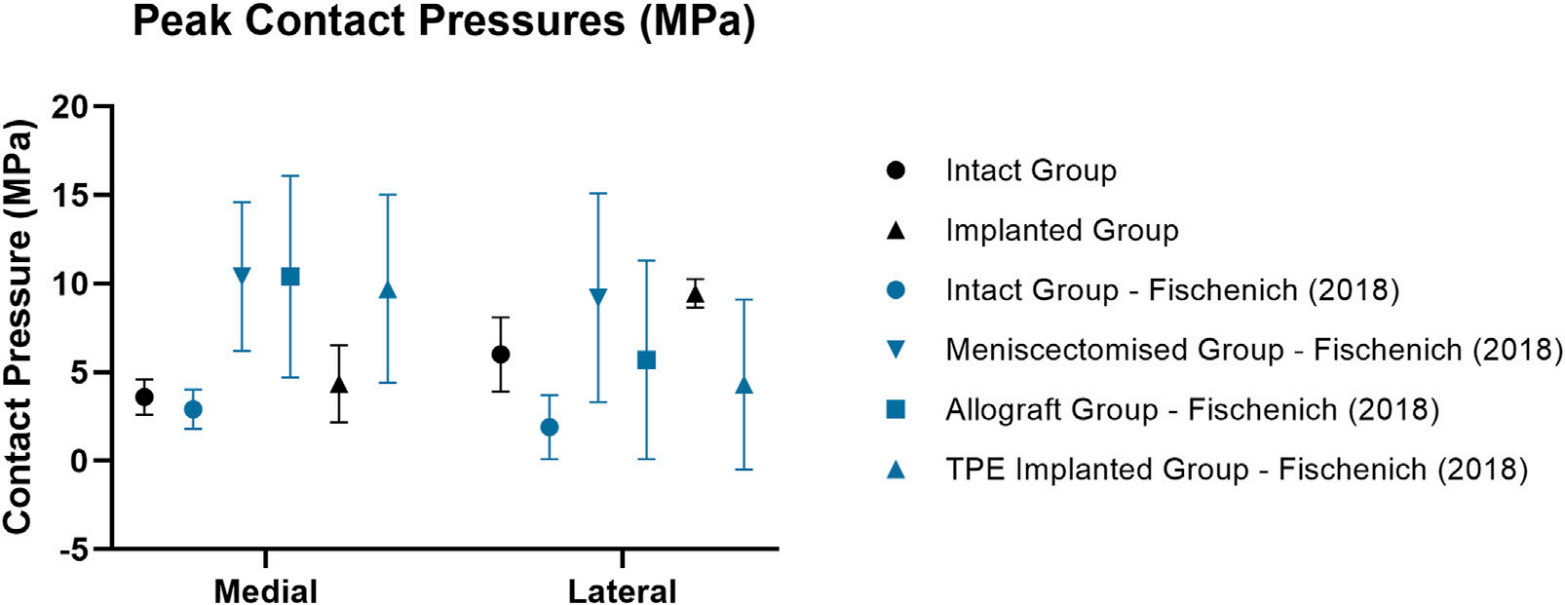
Mean ± standard deviation peak contact pressures (MPa) in the medial and lateral compartments of intact (circle), meniscectomized (downward arrow), allograft (square) and implanted (upright arrow) ovine stifle joints as measured in the current work (black) and in literature (blue). All Fischenich et al. (2018) contact pressure conditions were measured at 60° flexion with an axial load of 893N following meniscectomy, allograft transplantation or implantation in the medial compartment.

Medial peak pressures were not significantly different between the implanted and intact groups tested in this work (*p* > 0.4). The medial peak pressures of the implanted group were also not significantly different from the Fischenich et al. intact group (*p* > 0.1) and their TPE hydrogel implanted group (*p* > 0.08) (Figure 6). The medial peak pressures of the implanted group in the present study were significantly lower than the Fischenich et al. meniscectomized group (*p* < 0.03) and not significantly different from the Fischenich et al. allograft group (*p* > 0.09). These findings show that the fiber-matrix reinforced PVA-PEG hydrogel meniscal replacement restored the medial peak contact pressures.

Similarly to the intact group, the peak contact pressures of the implanted group were significantly higher in the lateral compartment (*p* < 0.01) following implantation in the medial compartment. This may have resulted from a change of limb alignment or joint congruity caused by the implantation procedure. In spite of this, the peak lateral contact pressures of the PVA-PEG hydrogel implanted group were not significantly different from the Fischenich et al. allograft group (*p* > 0.1) and TPE hydrogel implanted group (*p* > 0.08).

At 55° flexion, representing the stance phase of the gait cycle, peak contact pressures of the intact group were generally located on the posterior aspect of the medial tibial plateau, whereas the lateral peak contact pressures were located along the midline of the joint. These results matched those of previous studies (Heckelsmiller et al., 2017; Fischenich et al., 2018). However, Lee-Shee et al. found that peak contact pressures were located along the midline of the joint in both the medial and lateral compartments of the stifle, which could be a result of the applied constraints during testing (Lee-Shee et al., 2007). For the implanted group, peak contact pressures had shifted to the central aspect of the medial condyle at the cartilage-cartilage contact area and were distributed across the central aspect of the lateral condyle at 55° flexion. It could be speculated that the medial pressure distribution shift resulted from the absence of peripheral attachments on the ovine implant such as the meniscotibial ligaments and the deep fibers of the MCL (Allen et al., 1998) respectively, that constrain its anterior-posterior movement during knee flexion. Similarly, the effects of the surgical procedure and differences in conformity between the implant and each stifle joint on the contact pressure distributions cannot be ruled out and are not well understood. Nevertheless, the contact pressure distributions of the implanted group of this study were in agreement with those reported by Fischenich et al. (2018) for both the allograft and TPE hydrogel implanted groups in both compartments of the knee.

The kinematics of the ovine stifle in this study did not differ significantly from those reported *in vivo* (Tapper et al., 2006) in any of the degrees of freedom of motion. The general patterns of motion were the same, with coupled internal rotation and anterior translation of the tibia as the stifle flexed, and minimal movements in the remaining degrees of freedom. Thus the relatively simplified loading method via the distal tibia was adequate to replicate physiological gait and loading parameters reported previously (Taylor et al., 2006; Taylor et al., 2011). It would have been possible to record the joint kinematics at the beginning as well as the end of the test, but the lack of visible joint surface erosion meant that we did not suspect any resulting kinematic changes. Higher variability *in vivo* could be due to normal inter-subject gait variations, which did not occur in this controlled *in vitro* study. To the authors’ knowledge, this is the first study to assess 3D *in vitro* kinematics of intact ovine stifle joints in a physiological gait simulator, and to show that the passive ligaments and articular contacts guided the joint kinematics such that the motion was a close simulation of normal gait.

This study has limitations: firstly, tests were performed in a short timescale, under a consistent pattern of gait and with continuous motion, and did not fully simulate *in vivo* conditions, including dynamic muscle function and natural lubrication of a synovial joint. Joint simulators normally apply a consistent loading/kinematic profile to simulate normal gait to test wear of joint replacements. Variations in the pattern are not usually imposed by the control system. However, the novel ovine gait simulator uses native joints rather than prostheses, and they have inherent natural variability between specimens. The simulator enables the natural kinematics of the stifle joint because the kinematics are partly controlled by the ligaments, introducing tibial rotation and AP translations, so that the articulating path is not a linear one but also includes shear. Thus, there are inter-specimen variations in the gait pattern, but not simulation of different activities. Secondly, the ovine simulator operated at refrigeration temperatures of 4°C–8°C, which are significantly lower than body temperature (39°C) and could possibly affect friction and wear during joint articulation (Bortel et al., 2015). Lower testing temperatures were required to delay tissue necrosis across each 4-day long test, in order to obtain 500,000 cycles at physiological rates. Increasing the loading frequency would be unphysiological and would likely alter the lubrication regime, so the reduction of testing temperature was taken to be the least-bad compromise necessary to attain 500,000 cycles, equivalent to approximately a year of normal use by adult sheep *in vivo* (Ruckstuhl, 1998). Thirdly, contact pressures were limited to static measurements within the pressure film range (2.5–10 MPa), which allowed for the measurement of peak pressures but resulted in an incomplete contact pressure map because pressures below 2.5 MPa were not recorded. Peak contact pressures were prioritized in this study as they are a primary variable affecting chondroprotection and joint preservation. Furthermore, pressure film measurements are sensitive to temperature, humidity and shear forces, similarly to pressure sensors (Lee-Shee et al., 2007; Herregodts et al., 2015). The addition of plastic wrap, which was used to prevent its exposure to joint fluid, increased the pressure film thickness (240 µm thick) which could affect stifle joint contact mechanics (Herregodts et al., 2015).

The current feasibility study of the use of the ovine gait simulator to evaluate meniscus replacements was limited by a relatively small sample size (*n* = 5/6). Variability in the stifle joint anatomy, surgical procedure and stifle joint alignment in the ovine gait simulator (Houtem et al., 2006; DesJardins et al., 2007) warrant a larger sample size and a repeated-measures analysis in future work to obtain more definitive results when using the ovine gait simulator. Nonetheless, the results from this study had better repeatability than comparable ovine cadaveric studies reported in literature (Fischenich et al., 2018). Cartilage damage assessment across intact, meniscectomised and implanted conditions, could also be of interest and would require sourcing stifle joints from young sheep with pristine cartilage, which we leave for future work. Furthermore, a power analysis was not performed because this is a feasibility study to demonstrate the novel ovine gait simulator, rather than a study of the surgical procedures.

This study found that peak contact pressures and kinematics of intact stifle joints in the ovine gait simulator were comparable to literature, thus providing support for its efficacy as a tool to evaluate novel meniscus or other surgical procedures. The pilot study with the novel fiber-matrix reinforced meniscal replacement implant found that it restored medial peak contact pressures and native joint kinematics but did not restore lateral peak contact pressures. These findings support the use of an ovine gait simulator as a screening tool prior to evaluation of novel procedures in live animals.

## Data Availability

The original contributions presented in the study are included in the article/Supplementary Material, further inquiries can be directed to the corresponding author.

## Abbreviations

BCS, Bovine Calf Serum; BW, Body Weight; PMMA, Polymethyl methacrylate; PVA-PEG, Polyvinyl alcohol-polyethylene glycol; TPE, Thermoplastic elastomer.
